# The Relation of Attitude Toward Technology and Mastery Experience After an App-Guided Physical Exercise Intervention: Randomized Crossover Trial

**DOI:** 10.2196/28913

**Published:** 2022-02-18

**Authors:** Kai Sassenberg, Inka Roesel, Gorden Sudeck, Katharina Bernecker, Jennifer Durst, Inga Krauss

**Affiliations:** 1 Social Processes Lab Leibniz-Institut für Wissensmedien Tübingen Germany; 2 School of Science University of Tübingen Tübingen Germany; 3 Department of Sports Medicine University Hospital Tübingen University of Tübingen Tübingen Germany; 4 Institute for Clinical Epidemiology and Applied Biostatistics University of Tübingen Tübingen Germany; 5 Institute of Sports Science University of Tübingen Tübingen Germany; 6 Interfaculty Research Institute for Sports and Physical Activity University of Tübingen Tübingen Germany; 7 Department of Psychology University of Zurich Zürich Switzerland

**Keywords:** mobile app, exercise, mastery experience, self-efficacy, attitudes toward technology, osteoarthritis

## Abstract

**Background:**

Physical exercise has been found to assert a positive impact on many muscular conditions. Exercise under face-to-face supervision is the gold standard, but access to it is limited, for instance, for economic reasons. App-guided therapy is an intervention that is more affordable and easily accessible. However, attitude toward technology is a key predictor for media adoption and is therefore expected to shape user experience during app-guided therapy. This might be of particular importance for mastery experience, which is crucial for promoting exercise-related self-efficacy and perceived usefulness of the interaction. Both should empower patients to continuously exercise.

**Objective:**

This study sought to test whether attitudes toward technology predict mastery experience and perceived usefulness of the interaction after an app- versus a physiotherapist-guided treatment. We expect that attitudes toward technology positively predict both outcomes in case of the app-guided but not in case of the physiotherapist-guided treatment.

**Methods:**

Patients (n=54) with clinically diagnosed hip osteoarthritis participated in 2 training sessions with the same exercise intervention, once guided by an app on a tablet computer and once guided by a physiotherapist in a German university hospital. The order of the sessions was randomized. Attitude toward technology was assessed as predictor before the first session, while mastery experience and the global perceived usefulness of interaction as self-reported outcomes after each session.

**Results:**

In line with our hypotheses, attitude toward technology predicted mastery experience (b=0.16, standard error=0.07, *P*=.02) and usefulness of interaction (b=0.17, standard error=0.06, *P*=.01) after the app-based training but not after the training delivered by a physiotherapist (*P*>.3 in all cases). Mastery experience was lower for the app-based training but reached a very similar level as the physiotherapist-guided training for those holding a very positive attitude toward technology.

**Conclusions:**

The attitude toward technology predicts the extent of mastery experience after app-guided exercise therapy. As mastery experience is highly important for self-efficacy and future exercise behavior, attitudes toward technology should be considered when delivering app-guided exercise treatments.

**Trial Registration:**

German Clinical Trials Register DRKS00015759; https://www.drks.de/drks_web/navigate.do?navigationId=trial.HTML&TRIAL_ID=DRKS00015759

## Introduction

### Background

According to the World Health Organization (WHO), musculoskeletal conditions are the leading contributor to disability worldwide [1]. Their prevalence increases across the lifetime. For many of these conditions such as osteoarthritis (OA), physical activity and exercise contribute to the reduction of symptoms [2]. Therefore, physical exercise is recommended in many treatment guidelines [3-6]. Unfortunately, many patients do not follow these guidelines [7]. A reason among others is the fear of deterioration of their symptoms due to an incorrect execution of the exercises [8,9]. In other words, low self-efficacy is a major barrier for physical exercise.

This barrier can be overcome by supervision [10,11]. Exercises should initially be instructed by a health or exercise professional [5]. An alternative cost-efficient means to provide guidance regarding physical exercise is via digital apps on tablet computers. As tablets are highly mobile, they can be conveniently used in locations that allow to exercise [12-16]. They have a screen of sufficient size for video-based instructions and most importantly, older people are more likely to use tablets than smartphones [17]. However, the attitude toward digital technologies and with it the willingness of older adults to adopt digital technology for health purposes vary [18,19].

Therefore, this study sought to compare an app-based intervention with the gold standard of an intervention supervised by a physiotherapist. We tested the effect of both treatments on *mastery experience*, which is known to facilitate exercise-related self-efficacy and, in turn, continuous exercising [20]. In addition, we studied the perceived *usefulness of the interaction*, a key predictor of the attitudes toward the intervention which should also be related to continuous exercise [21]*.* To conduct a fair test, both interventions rely on the same evidence-based exercise intervention for patients with hip OA [22,23]. To do justice to the older age of the target group of this intervention and the varying acceptance of technology-based health interventions in that group, we considered attitudes toward technology as an additional predictor.

### Theoretical Underpinning of (Digital) Exercise Interventions

The model of physical activity–related health competence (PAHCO) [24,25] guided the development of the examined app as well as this study. The core idea of this health education model is that exercising in a health-effective and low-risk manner requires a set of competences, namely, movement competence, control competence, and self-regulation competence. *Movement competence* includes motor abilities and skills as well as movement and body awareness. To train this competence, we ensured that our app provided detailed instructions regarding movement as well as body signals and allowed for the repeated viewing of videos until instructions were well understood. *Control competence* requires activity-related knowledge and the ability to perceive and interpret body signals (eg, to sense muscle soreness and adjust exercise intensity based on it). To direct users’ attention to this aspect, the app contained questions about pain and intensity after each exercise and provided feedback on how to adapt the exercise to ensure optimal dose parameters.

Finally, *self-regulation competence* summarizes motivational and volitional determinants of regular exercise including self-efficacy, which refers to the feeling that exercise can be executed independently and, thus, key for its uptake [26]. Self-efficacy is developed through the experience that the exercise session empowers the user to execute the exercises effectively, called *mastery experience* [20,27]. Mastery experience can refer to the movement-related demands (which relates to movement competence), the self-directed control of physical loads (which relates to control competence), or—most relevant in the current context—the app- or physiotherapist-guided exercise instructions. Given that mastery experience is decisive for the adoption of regular exercise [26,28] and the use of digital devices more generally [29], we focused particularly on this indicator in this study.

### Attitudes Toward Technology Among Older Adults

Attitudes toward technology and acceptance of eHealth vary substantially among older adults [30,31]. Research has demonstrated that the general attitude toward technology relate positively to the judgment of health-related technologies including (1) the perceived usefulness and (2) the self-efficacy regarding the use of the specific technology [32]. This suggests that attitudes toward technology might relate to perceived usefulness and self-efficacy, because the attitudes color the experience during technology use, including perceived usefulness of the interaction and mastery experience. Given that perceived usefulness and mastery experience both contribute substantially to the adoption of the technology [21,33] and, thus, in the current context to health behavior, knowledge about the relation between these variables is highly relevant. At the same time, there is no reason to assume that attitudes toward technology predict the usefulness of the interaction and the mastery experience in the context of interventions delivered by a human instructor. Accordingly, we hypothesized:

Attitudes toward technology is positively related to (1) the mastery experience and (2) the perceived usefulness of the interaction regarding an app-guided treatment but not regarding a treatment delivered by a human instructor.

### The Current Research

These hypotheses were tested using the data collected in a larger training study, parts of which have been reported by Durst et al [34]. In this experimental study, patients with hip OA received the same evidence-based exercise intervention [22,23] once delivered by an app on a tablet computer and once by a physiotherapist with the order of sessions being randomized between participants. Attitudes toward technology, mastery experience, and usefulness of the interaction were assessed after both sessions.

## Methods

### Design and Participants

Parts of this section correspond to those of a previous publication on this study [34] given that both articles describe the same study. We conducted a randomized crossover trial (see Multimedia Appendix 1 for the CONSORT checklist) with a 2 (treatment: app vs physiotherapist—within participants) × 2 (sequence—between participants) design. The attitude toward technology was assessed as additional continuous predictor. Participants were randomly assigned in a 1:1 allocation ratio to the 2 exercise treatment sequences. Randomization was based on a list generation with an online tool [35].

The AP (app–physiotherapist) group first had a training session using a tablet computer–based app and later a second session supervised by a physiotherapist, whereas the PA (physiotherapist–app) group was supervised by the physiotherapist in the first session and had the app-based training in the second session. For each participant the 2 intervention sessions were scheduled 4-6 weeks apart to allow for a sufficiently strong washout of treatment effects. The analyses reported by Durst et al [34] show that washout was only partly successful regarding movement competence. Therefore, we include sequence as a factor in the analyses reported below. Ethical approval for this study was obtained from the Ethical Committee of Tuebingen University Hospital. The study was registered in the German Clinical Trials Register (DRKS00015759). This preregistration did not include the hypothesis tested here.

Participants with diagnosed hip OA were recruited via advertisements in regional newspapers, by an email sent out via the employee list-serve of the University of Tuebingen and the Tuebingen University Hospital, and via flyers distributed by orthopedic surgeons and physiotherapists. In a telephone call interested individuals were screened for eligibility (for exclusion criteria, see Textbox 1). Eligible people were then randomly allocated to 1 of the 2 treatment sequences (determined by the next free slot in the randomization list) and informed about (1) the positive effects of exercise therapy for hip OA, (2) the details of the treatment, and (3) the research questions. Finally, the 2 treatment sessions at the Tuebingen University Hospital were scheduled.

**Inclusion and exclusion criteria.**

**Inclusion criteria**

1. 50 years and older
2. Self-reported lifetime prevalence of hip osteoarthritis diagnosed by a medical practitioner
3. Informed consent to study participation

**Exclusion criteria**

1. Comorbidities leading to major impairments in everyday life and representing contraindications for physical activities
2. Self-reported acute illness
3. Significantly established osteoporosis requiring treatment, previous spontaneous or low-impact fracture
4. Musculoskeletal surgery at the lower extremity within the last 3 months
5. Regular use of gait aids (eg, walker, crutch)
6. Insufficient German language skills for self-administered questionnaires
7. Previous experience from hip exercise groups

**In case of an artificial joint replacement at the other hip or the knee joints:**

1. Artificial joint replacement at the knee or hip joint or both within the last 6 months, with unstable anchoring or with known radiological signs of implant loosening
2. Current pain at rest or with activity due to artificial joint replacement
3. Luxation as an adverse event of artificial hip replacement
4. Acute joint inflammation at the knee or hip joint or both

### Trial Interventions

#### Overview

The interventions (physiotherapist and app) used in this study were extracted from an evidence-based 12-week exercise program that was specifically designed for patients with hip OA [23,36,37]. Four exemplary exercises and their instructions were selected from this program. Both types of training sessions lasted 45-60 minutes. Participants were asked to report perceived exertion and OA-related pain after each set using a 10-point Likert scale.

#### Physiotherapist-Guided Exercises

The physiotherapist had 5 years of work experience. She introduced the exercises, corrected deficient or improper execution, and asked to adjust the exercise to the planned level of intensity, and in case of increasing pain according to the used target values of physical exhaustion and pain that had been implemented in the algorithm of the app to modify exercise intensity instructions. The physiotherapist also adapted the intensity level for the participant on an individual basis, as applicable.

#### App-Guided Exercises

The app was designed for and presented on a 9.7-in. (24.64 cm) tablet computer, which was mounted on a holder in a convenient position. All instructions were given on the tablet, after the app had been started by the experimenter. In line with the PAHCO model, the app supports practical exercises, cognitive and motor learning, and the processing of personal experience with movement [38]. The app consists of 5 components: (1) technical introduction, (2) creation of an individual user profile, (3) pedagogical agent, (4) exercise introductions, and (5) feedback-based dose adjustments and further instructions. Videos and acoustic signals are implemented in the software to guide the different exercises and to support the participant during the exercises. The videos combine long shots and close-ups based on interviews in a pretest. In addition, the camera’s perspective and the choice of actors were optimized based on the results of the pretest to render the starting position and the movements easily visible. Movement speeds for exercise repetition are set using an auditory signal and visually supported by the actor in the video. For details about the elements and the algorithms of the app, see Multimedia Appendix of Durst et al [34].

### Measures

#### Sample Characteristics

Sociodemographic, anthropometric, personal, OA-related variables, and additional measures unrelated to the current research question were assessed before the first training session.

#### Attitudes Toward Technology

A validated 19-item scale for attitudes toward technology (German: “Technikaffinität” TA-EG [39]) was presented before the first training session. Participants had to indicate their agreement to each item (eg, “I enjoy trying out electronic devices”; *α*=.83) on a 5-point scale (1=*does not apply at all*, 5=*exactly applies*). Ratings were averaged and summarized in one index by averaging the values after recoding negatively worded items. Higher values indicate a more positive attitude toward technology (see Table 1 for descriptive statistics).

**Table 1:** Baseline data for the complete sample differentiated according to treatment sequence.

Characteristics		Total (n=54)	PA^a^ (n=26)	AP^b^ (n=28)	*P* value
Age (years), mean (SD)		62.4 (8.2)	62.5 (8.0)	62.3 (8.5)	.91
**Gender**					.74
	Female, n (%)	32 (59)	16 (62)	16 (57)	
	Male, n (%)	22 (41)	10 (39)	12 (43)	
**Education**					.19
	Academic education, n (%)	22 (41)	8 (31)	14 (50)	
	Vocational education, n (%)	31 (57)	18 (69)	13 (46)	
	No vocational education, n (%)	1 (2)	0 (0)	1 (4)	
**Work situation**					.44
	Employed, n (%)	32 (59)	14 (54)	18 (64)	
	Retired, n (%)	22 (41)	12 (46)	10 (36)	
Experience with exercise groups (1-5), median (IQR)		3.00 (1.0)	3.00 (1.0)	3.00 (2.0)	.31
Daily everyday activity (minutes of cycling and walking/week), median (IQR)		215 (360)	215 (330)	225 (458)	.49
Sports activity (minutes/week), median (IQR)		209 (273)	229 (309)	184 (308)	.26
Attitudes toward technology, median (IQR)		3.16 (0.5)	3.13 (0.5)	3.20 (0.5)	.63

^a^PA: physiotherapist–app.

^b^AP: app–physiotherapist.

#### Mastery Experience and Perceived Usefulness of the Interaction

Mastery experience and perceived usefulness of the interaction were each measured once for the physiotherapist and once for the app. Four items were used to assess the *mastery experience* regarding the exercise after each session, of which 2 were adopted from the subscale *Competence* of the Need Satisfaction in Exercise Scale [40] (eg, “I had the impression that I was executing the exercise effectively”; internal consistency: *α*_APP_=.88; *α*_PHYSIO_=.67). Four additional items assessing the *usefulness of the interaction* were self-developed and 1 was adopted from the usability measure by Harder et al [41] (eg, “The instructions were helpful”; *α*_APP_=.85; *α*_PHYSIO_=.54). The internal consistency for the usefulness of the interaction with the physiotherapist was not satisfying. Given that it could not be improved by dropping an item and that we aimed at parallel measures for both interventions, we did use the scale nonetheless. Both scales used a 4-point scale (1=*does not apply at all*, 4=*exactly applies*; see Table 2 for descriptive statistics). For all items, see Multimedia Appendix 2.

**Table 2:** Mean (SD) of mastery experience and usefulness of interaction by sequence and treatment.

Measure		Total (n=54)	PA^a^ (n=26)	AP^b^ (n=28)
**Usefulness of interaction**				
	Physio (n=49)	3.84 (0.24)	3.84 (0.27)	3.84 (0.22)
	App (n=51)	3.32 (0.68)	3.43 (0.57)	3.22 (0.75)
**Mastery experience**				
	Physio (n=49)	3.51 (0.32)	3.55 (0.27)	3.47 (0.36)
	App (n=51)	3.16 (0.56)	3.34 (0.40)	3.00 (0.64)

^a^PA: physiotherapist–app.

^b^AP: app–physiotherapist.

### Sample Size

We planned to collect data from at least 40 participants.

### Statistical Analysis

Participant characteristics are summarized for the whole sample and for the 2 sequence conditions (Table 1). We tested for differences between sequence conditions using Pearson chi-square test for categorical data, independent Student *t* test for indices from rating scales, or Mann–Whitney *U* test. The latter was used if the assumption of normally distributed data was violated.

The main hypothesis was tested using a linear mixed design analysis of variance (mixed analysis of variance) with participant as random factor (nested within sequence of treatment order) and treatment (P and A), sequence (PA and AP), period (T1 and T2), and attitude toward technology (mean centered) as well as their (2- and 3-way) interactions as fixed factors separately for mastery experience and usefulness of interaction. Effects of interactions were resolved using simple slope analyses. We report the results based on analyses assuming normal distribution of the variables. In cases where this assumption was violated, we repeated the analyses after normalization of scores and these scores were used for further data analysis. Results of both analyses were virtually identical.

The level of statistical significance was set at the conventional level of *α*=.05. All data were analyzed using SPSS version 25 (IBM) and R version 3.6.1 (The R Foundation).

## Results

### Participants

**Figure 1:**
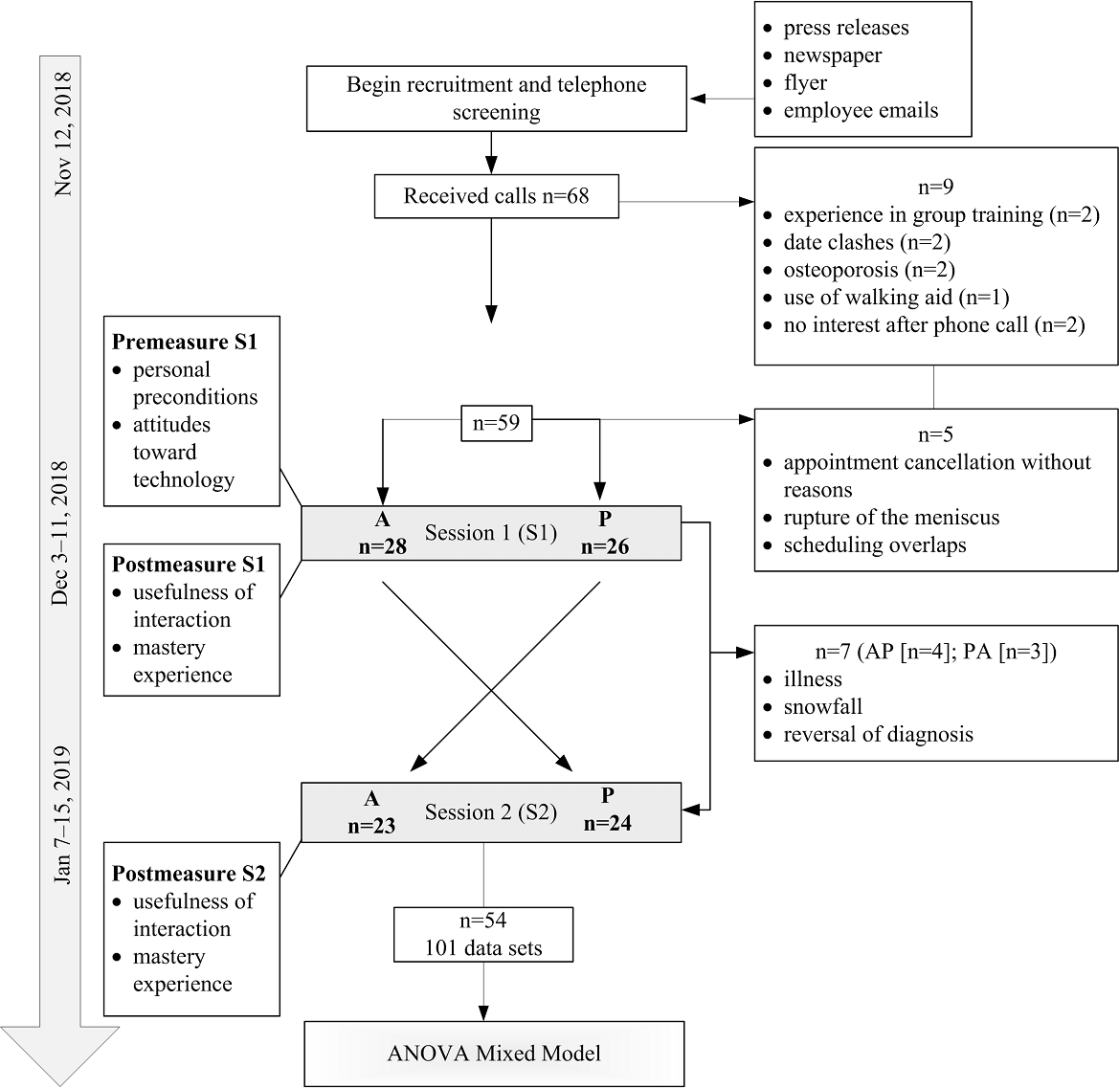
Study flowchart. A: app; AP: app-guided followed by a physiotherapist-guided sequence; P: physiotherapist; PA: physiotherapist-guided followed by an app-guided sequence.

Among 68 people, 59 fulfilled our inclusion criteria, contacted the study staff, and made an appointment. Five individuals canceled the first training appointment. Of the remaining 54 participants who completed the first training session, 7 could not attend the second session. One participant did not provide the ratings of the physiotherapist in the first session. Therefore, this case drops out of all analyses including this measure. Further details on flow of participants are depicted in Figure 1. The individual period between T1 and T2 ranged from 27 to 42 days, with an average interval of 34.7 days.

### Baseline Data

The key baseline characteristics including physical activity and exercise-related experiences did not differ between participants allocated to the 2 treatment sequences (Table 1). For additional information, see Durst et al [34].

### Hypothesis Testing

The analysis for *mastery experience* revealed a main effect of treatment, *F*_1,41.9_=14.89, *P*<.001, *η²*_part_=0.26, CI_b-90%_ of 0.09-0.43, which was again qualified by the expected treatment × attitudes toward technology interaction, *F*_1,41.6_=5.95, *P*=.02, *η²*_part_=0.12, CI_b-90%_ of 0.01-0.29. In addition, there was a main effect of sequence factor, *F*_1,42.8_=4.16, *P*=.05, *η²*_part_=0.09, CI_b-90%_ of 0.00-0.24. In the PA condition the mastery experience was perceived more positive across both treatments than in the AP condition, which is mostly driven by the judgment of the app (Table 2). The other main and interaction effects were not significant (in all cases, *F*<3.6 [41<*df*<43], P>.05).

Similarly, the analysis for *usefulness of interaction* revealed a main effect of treatment, *F*_1,42_=26.98, *P*<.001, *η²*_part_=.38, CI_b-90%_ of 0.20-0.54, which was qualified by the predicted treatment × attitudes toward technology interaction, *F*_1,41.7_=4.88, *P*=.03, *η²*_part_=0.10, CI_b-90%_ of 0.00-0.26. All other main effects or interactions were not significant (in all cases, *F*<2.2 [41<*df*<43], *P*>.10).

**Figure 2:**
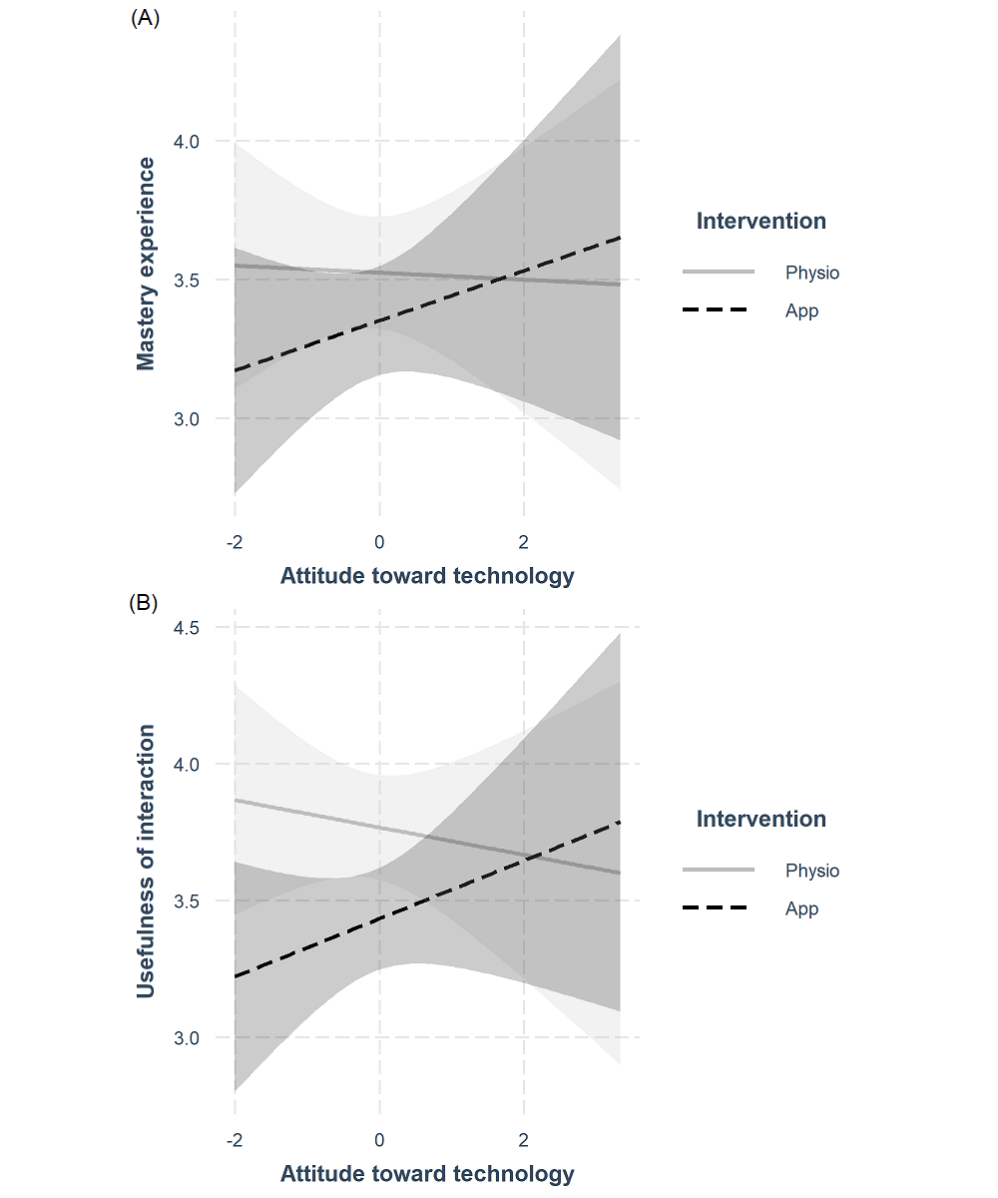
(A) Usefulness of interaction and (B) mastery experience by attitudes toward technology and intervention. Shaded areas represent 95% CIs.

Simple slope analyses revealed that a more positive attitude toward technology correlated with a more positive mastery experience, *b*=0.16, standard error=0.07, *t*_43_=2.46, *P*=.02, CI_b-95%_ of 0.03-0.29, and a higher usefulness of the interaction, *b*=0.17, standard error=0.06, *t*_43_=2.76, *P*=.01, CI_b-95%_ of 0.05-0.30, regarding the app-based intervention, but not regarding the physio, both │*t*_43_│<1 and *P*>.3 in all cases (Figure 2).

## Discussion

### Principal Findings

This study aimed at investigating the role of attitudes toward technology for the development of PAHCO (ie, mastery experience and the usefulness of the interaction) comparing app- and physiotherapist-guided exercise. We hypothesized that attitudes toward technology would predict the mastery experience and the perceived usefulness of the interaction regarding app-guided exercise but not regarding physiotherapist-guided exercise. The results supported this prediction.

Overall mastery experience and usefulness of the interaction were lower as an outcome of app-guided exercise than as an outcome of physiotherapist-guided exercise. However, this main effect of intervention type was qualified by the predicted attitudes toward technology × intervention type interaction. For people with a less positive attitude toward technology both outcomes were lower after the app-guided intervention than after the physiotherapist-guided intervention. This difference was substantially reduced for people with more positive attitudes toward technology and descriptively disappeared 2 SD above the mean, suggesting that only a few people with a very positive attitude toward technology might benefit to a similar extent from an app-based intervention as from a physiotherapist-guided intervention (but see the “Limitations” section). It should be noted, however, that as reported in Durst et al [34] the movement performance (at least for more complex exercises) is higher after physiotherapist-guided exercise compared with app-guided exercise.

Consistent with a recent review mainly referring to qualitative studies [42], our quantitative study approach provides additional evidence for the importance of attitudes toward technology in the process of implementing app-guided exercise interventions (and potentially also health apps including other interventions). People holding a less positive attitude toward technology benefit less in their health competence from the use of an app-guided intervention. This will not only work against the persistent use of such apps but also undermine the long-term health benefits that using such an app could have. In an environment where policy makers stress the self-reliance of patients and a rapidly growing amount of health technologies become available (and partly also replace other interventions), this is an important finding to be considered. Those holding a less positive attitude toward technology might face disadvantages. One intervention that might help to increase positive responses to app-based interventions among those holding a less positive attitude toward technology is a session in which the app is introduced face-to-face. This might result in increased self-efficacy, and therefore most likely also app use. Thus, combining the app with a face-to-face intervention might prevent disadvantages of those with negative attitudes toward technology that might otherwise occur.

What might drive the effects of attitudes toward technology? We assume that people with a negative attitude focus on different (ie, more negative) experiences while using a new technology than people holding a positive attitude. This attention-based explanation effect is speculative and should, thus, be tested in future research.

### Limitations

This study has some limitations that should be noted. The means for the outcome measures after both interventions, but in particular after the physiotherapist-guided intervention, are very high. We are, thus, potentially dealing with a ceiling effect for both outcomes. This will most likely lead to an underestimation of effect sizes. Moreover, the intersections between regression lines should be interpreted with caution. Further research with larger samples and measures capturing the variance in the upper range of the scale in a more differentiated manner should be conducted before drawing conclusions about the level of attitudes toward technology from which an equality of both interventions could be assumed.

The usefulness of interaction scale for the physiotherapist had a low internal consistency, but analysis based on single items do not result in a different pattern compared with the reported analysis. This indicates that the current results are stable even though the internal consistency of 1 indicator was low. The low internal consistency most likely results from the richer impression formation process for humans than for technology. The more differentiated impression people have about the physiotherapist might have contributed to a lower correlation between the aspects summarized in the usefulness of the interaction with the physiotherapist scale (compared with the app). At the same time, similar scales are required to compare the outcomes of both types of intervention. Future research might opt for a more differentiated measurement approach making up for this issue.

### Strengths

Confronting each participant with both the app- and physiotherapist-guided intervention in randomized order is a strength of this study. It should, however, be noted that this might lead to carryover effects, that is, the outcome of the second intervention might be affected by the first intervention. In the reported analysis, the relevant sequence × treatment interactions were not significant. However, for transparency reasons we would like to note that the attitudes toward technology × treatment interactions are descriptively stronger when the app is presented first. If this difference is replicated in future research, it would indicate that the attitude toward technologies is less relevant and the use of exercise apps is particularly beneficial after exercise sessions guided by a physiotherapist—for instance, as a refresher or as an extension. The results of the movement performance data point in the same direction [34].

A further strength of this study is that we compared the outcomes of using an exercise-related app with the gold standard of a physiotherapist-guided exercise, whereas many studies only focus on the evaluation of apps (often comparing it with a no intervention control condition or paper instructions only). Our comparison sets a very high standard and in this light the difference between both interventions is not surprisingly high. This might in part result from another strength of this study, namely the fact that the exercise program implemented in the app is an evidence-based exercise intervention [22,23]. Finally, it should be noted that the study was conducted among diagnosed patients of an age group that is usually considered as being less technology savvy.

### Conclusion

This study provided evidence for the impact of attitudes toward technology for the outcomes of app-guided but not of physiotherapist-guided physical exercise interventions regarding PAHCO. A positive attitude toward technology predicted higher mastery experience after an app-guided but not after a physiotherapist-guided intervention, which is most likely beneficial for task self-efficacy. Therefore, attitudes toward technology should be considered when prescribing and implementing app-based interventions to ensure task self-efficacy and beneficial effects on competencies for a healthy, physically active lifestyle.

## Appendix

Multimedia Appendix 1 CONSORT-eHEALTH checklist (V 1.6.1).

Multimedia Appendix 2 Items assessing mastery experience and usefulness of interaction.

## Abbreviations

AP: app–physiotherapist
OA: osteoarthritis
PA: physiotherapist–app
PAHCO: physical activity–related health competence
WHO: World Health Organization
