# Supplementary material for: The Relation of Attitude Toward Technology and Mastery Experience After an App-Guided Physical Exercise Intervention: Randomized Crossover Trial
Source: JMIR Form Res. 2022 Feb 18;6(2):e28913. doi: 10.2196/28913 (PMC8900907; doi:10.2196/28913)
Supplement: Multimedia Appendix 2 [file formative_v6i2e28913_app2.docx]

**Multimedia Appendix 2**. Items assessing mastery experience and usefulness of interaction.

*Items assessing mastery experience:*

I had the feeling of being very competent for the activities.

I had the impression that I was executing the exercise effectively.

The instructions and guidance gave me confidence in performing the exercises.

I had the impression that I could optimally adapt the exercises to my personal conditions.

*Items assessing usefulness of interaction*

The trainer/the app kept me motivated throughout the duration of the training.

The instructions were helpful.

The instructions were customized to my needs.

I felt that the trainer/the app enabled me to perform the exercises correctly even at a later stage without assistance.
